# Habituation Training Improves Locomotor Performance in a Forced Running Wheel System in Rats

**DOI:** 10.3389/fnbeh.2017.00042

**Published:** 2017-03-08

**Authors:** Angel Toval, Raúl Baños, Ernesto De la Cruz, Nicanor Morales-Delgado, Jesús G. Pallarés, Abdelmalik Ayad, Kuei Y. Tseng, Jose L. Ferran

**Affiliations:** ^1^Department of Human Anatomy and Psychobiology, School of Medicine, University of MurciaMurcia, Spain; ^2^Institute of Biomedical Research of Murcia (IMIB), Virgen de la Arrixaca University Hospital, University of MurciaMurcia, Spain; ^3^Department of Physical Activity and Sport, Faculty of Sport Science, University of MurciaMurcia, Spain; ^4^Human Performance and Sports Science Laboratory, University of MurciaMurcia, Spain; ^5^Department of Cellular and Molecular Pharmacology, The Chicago Medical School at Rosalind Franklin UniversityNorth Chicago, IL, USA

**Keywords:** physical activity, exercise, rodents, familiarization protocols, acclimation protocols

## Abstract

Increasing evidence supports that physical activity promotes mental health; and regular exercise may confer positive effects in neurological disorders. There is growing number of reports that requires the analysis of the impact of physical activity in animal models. Exercise in rodents can be performed under voluntary or forced conditions. The former presents the disadvantage that the volume and intensity of exercise varies from subject to subject. On the other hand, a major challenge of the forced training protocol is the low level of performance typically achieved within a given session. Thus, the aim of the present study was to evaluate the effectiveness of gradual increasing of the volume and intensity (training habituation protocol) to improve the locomotor performance in a forced running-wheel system in rats. Sprague-Dawley rats were randomly assigned to either a group that received an exercise training habituation protocol, or a control group. The locomotor performance during forced running was assessed by an incremental exercise test. The experimental results reveal that the total running time and the distance covered by habituated rats was significantly higher than in control ones. We conclude that the exercise habituation protocol improves the locomotor performance in forced running wheels.

## Introduction

According to the World Health Organization (2010), the lack of physical activity is the fourth leading risk factor for global human mortality. For instance, reduced physical activity is associated with higher risks of developing obesity, type 2 diabetes, osteoporosis, depression and cardiovascular diseases (World Health Organization, 2010; Dishman et al., 2013; Mora-Rodriguez et al., 2016). On the other hand, there is growing evidence supporting a positive impact of increasing regular levels of physical activity on public health (Dishman et al., 2006; Hillman et al., 2008; van Praag, 2009; Vivar et al., 2012). These studies suggest that motor skill training and regular exercise are beneficial to sustaining proper executive functions of cognition and learning (e.g., motor learning in the spinal cord; Edgerton et al., 2004; Hillman et al., 2008), and in some cases it may confer protective effects against the onset of neurological disorders including Parkinson’s disease (Smith and Zigmond, 2003), Alzheimer’s dementia (Cotman and Berchtold, 2002) and stroke (Stummer et al., 1994). However, the neurobiological mechanisms associated with physical activity are not entirely known, partly due to a lack of uniformity and parameterization in experimental protocols employed to assess the impact of exercise in animal models. For example, rodent studies using running-wheels often employ protocols that allow *ad libitum* access to the wheel. While such an approach has its advantages, both the intensity and volume varies significantly from subject to subject due to the “voluntary” nature of the experimental design (van Praag et al., 2005; Kregel et al., 2006; Leasure and Jones, 2008; Creer et al., 2010; Kobilo et al., 2011; Marlatt et al., 2012). One way to overcome these challenges is to implement a forced running-wheel protocol in which the same training load is applied to all subjects (Auriat et al., 2006; Shimizu and Yamanouchi, 2011; Wang et al., 2013; Chen et al., 2014). It becomes clear from these studies that the inclusion of a pretraining stage of habituation prior to the testing phase is a crucial step to achieving better performances in response to increasing running demands (Dick, 2007). Thus, the aim of the present study is to develop and evaluate a protocol of habituation training to enhance the locomotor performance of young adult rats subjected to a progressive incremental running load test in a forced running-wheel system (Bentley et al., 2007).

## Materials and Methods

All experimental procedures were approved by the University of Murcia’s animal care and use committee according to the Spanish regulation (Royal Decree 1201/2005) and European Union Directive 2003/65/EC of the European Parliament (Amending Council Directive 86/609/EEC) guide for care and use of laboratory animals.

### Animals and Experimental Groups

Young adult male Sprague-Dawley rats (Laboratory Animals Facilities at the University of Murcia) were group housed (2–3 rats/cage) and kept in a 12:12 h light/dark cycle room (dark period from 8 AM to 8 PM) at 21–23°C and 55 ± 5% of relative humidity, with food and water available *ad libitum*. Rats were randomly assigned to receive either the protocol of habituation training (habituated) or not (non-habituated) for eight consecutive days. The locomotor performance to incremental intensities of forced running was assessed at 1, 3, 31 and 33 days post-last habituation session.

### Running-Wheel Training

**Figure 1:**
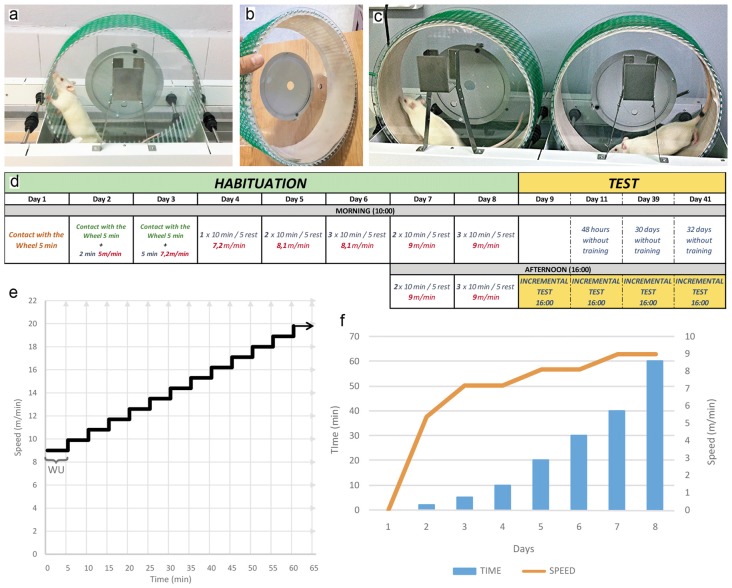
**(A)** Forced motor wheel with aluminum bars in the running surface. **(B)** Running surface of the wheel covered by denim fabric. **(C)** Forced motor wheel system during a running session. **(D)** The schedule shows the exercise program developed during the habituation exercise protocol. The speed, time and number of sessions by day are described. The incremental test is developed after 24 h finished the habituation, and repeated at 3, 31 and 33 days later. **(E)** This graphic is a representation of time variation (*X* axis) in relation to the speed variation (*Y* axis) during the development of the incremental exercise test. Notice that every 5 min the speed changes increasing in 0.9 m/m giving the aspect of steps. **(F)** Bars and lines graph which represents the training load in each day (*X* axis) of the habituation protocol. Bars indicate time of running (*Y* axis, left). Line indicates speed (*Y* axis, right). The training load, speed and time followed an upward progressive pattern. WU, warming up phase.

Six polycarbonate motor running-wheels were purchased from *Lafayette-Campdem* system (80805A model, dimensions 129.54 × 45.47 × 42.93 cm). The internal surface of the running-wheel was covered with custom-made denim fabric to provide a smooth flatten running surface (Figures 1A–C). The habituation phase is comprised of 10 sessions distributed across 8 days of training. During these sessions, both intensity (speed) and volume (time) were increased following an upward progressive pattern as summarized in Figures 1D,F. Once the habituation phase is completed, rats were subjected to the first incremental exercise test 24 h after the last habituation session. In order to determine whether the habituation sessions exert an enduring impact on forced running performance, rats were tested again at 3, 31 and 33 days from the last session of habituation training. During the testing phase of incremental forced running, a defined speed of 9 m/min is introduced at the beginning of each test followed by increments of 0.9 m/min every 5 min until a failure to maintain a running pattern becomes apparent (Figure 1E). Criteria to stop the incremental test include jumping, crawling and/or rolling within the running-wheel. Typically, rats are removed from the running-wheel and the test stopped if two or more consecutive uncontrolled laps are detected. The time spent in the running wheel was determined from the beginning of the test to its termination when the animal fails to maintain a running pattern.

### Statistical Analysis

All data were presented as mean ± standard error of the mean. A two-tailed student’s *t*-test was used for two-group comparison involving a single continuous variable and a one-way repeated measures analysis of variance (ANOVA) test was used for intra-subjects multiple comparison. Differences between the experimental groups were considered statistically significant at *P* < 0.05 (StatSoft, Tulsa, OK, USA).

## Results

Comercially available running-wheels were modified to include a custom-made denim fabric into the internal surface of the wheel to provide a smooth flatten running surface (Figures 1A–C). In order to assess the impact of habituation training, a cohort of young adult male Sprague-Dawley rats were subjected to a protocol of habituation comprised of 10 sessions in 8 days during which the intensity (speed) and the volume (time) of the training sessions were increased following an upward progressive pattern (Figures 1D–F). We observed a 100% success rate response, that is, all animals (*n* = 23) tested with this tranining protocol managed to complete the entire 8 days of habituation running.

**Figure 2:**
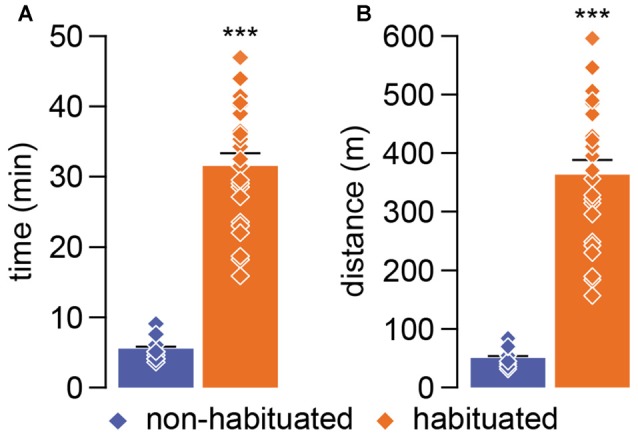
**(A)** The graph represents the total time of running endured during the incremental test comparing non-habituated (blue) and habituated (orange) young adult rats. Individual measures are indicated by diamonds and the mean comparison by bars (non habituated: *X* = 5.42 ± 0.5 min; habituated: *X* = 31.70 ± 1.8 min). **(B)** The graph represents the total distance covered during the incremental test comparing non-habituated (blue) and habituated (orange) rats. Individual measures are indicated by diamonds and the mean comparison by bars (non habituated: *X* = 49.57 ± 4.87 m; habituated: *X* = 368.29 ± 25.49 m). ***p < 0.0001; two-tailed student’s *t*-test.

We next developed a progressive incremental running load test (Figure 1E) to assess the impact of habituation training on locomotor performance. Relative to the non-habituated group (*n* = 12), rats that underwent the habituation training phase exhibited a six fold increase in performance to the incremental running load test as revealed by the time and distance spent in the wheel (Figure 2). While most of the animals from the non-habituated group failed to pass the first 5 min step of the incremental test, habituated rats run an average of ~32 min covering a mean distance of ~370 m when tested 24 h after the last habituation session (Figures 2A,B).

**Figure 3:**
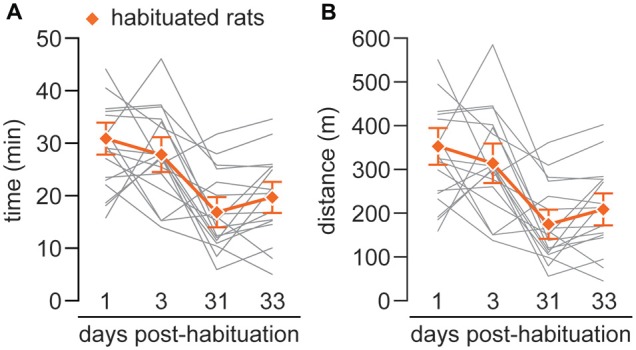
**(A)** The graph represents the total time of running of each individual (gray lines) and the mean of time (orange line) during the four incremental tests carried out at 1 day after the completion of the habituation phase and repeated after 3 (*X* = 27.9 ± 1.74 min), 31 (*X* = 15.81 ± 1.46 min) and 33 (*X* = 18.58 ± 1.54 min) days.** (B)** The graph represents the total distance covered during the four incremental tests for each individual (gray lines) and the mean of distance (orange line) of habituated rats after 1, 3 (*X* = 314.99 ± 24.2 m), 31 (*X* = 162.27 ± 17.29 m) and 33 (*X* = 194.95 ± 18.53 m) days. A comparison between tests at 1 and 3 day vs. performed at 31 and 33 day show statystically significant differences (*p* < 0.002; one-way repeated measures analysis of variance (ANOVA)).

Finally, the incremental test was repeated to evaluate long term effects of the habituation. The locomotor performance at 3 days remains similar; however it is significantly decreased both at 31 and 33 days (habituated rats run an average of ~16/18 min covering a mean distance of ~162/194 m; respectively; Figures 3A,B).

These results support that the habituation period improves the locomotor performance in forced running wheels; but this effect is progressively drecreased without a permanent training.

## Discussion

The present study was developed to evaluate the impact of an habituation training in the locomotor performance, using a forced running wheel system in young adult rats. It is known that wild rats cover long distances during the night, running bursts of short periods at high speeds (Tchernichovski and Benjamini, 1998). A similar pattern of running is detected in voluntary exercise laboratory paradigm, that allow rats to reach higher intensities and cover longer distances than in forced exercise; but restricting the posibility to manage intensity and volume of running (training load; Leasure and Jones, 2008). This situation highlights the difficulty of the voluntary running to do precise correlations between training load and the observed effects (van Praag et al., 2005; Leasure and Jones, 2008; Creer et al., 2010; Kobilo et al., 2011; Marlatt et al., 2012). On the other hand, in forced conditions, rats run during larger periods of time but at lower speed than observed during voluntary running (Narath et al., 2001; Leasure and Jones, 2008). Although forced models are consistent with the uniformity of the physical activity carried out for the group of rats, running on a treadmill or a motorized wheel can be a challenge for the animals. In fact, as many as 10% of the rats refuse to walk or run on a treadmill; and these animals must be removed from exercise studies (Jasperse and Laughlin, 1999; Koch and Britton, 2001; Kregel et al., 2006). Our results support that habituation exercise training are key to get better locomotor performances to develop succesful training programs in forced conditions.

Only a few of the current works in forced running paradigm consider the implementation of an habituation phase as a good strategy to reduce the number of rats classified as nonrunners (Kregel et al., 2006; O’Dell et al., 2007; Chen et al., 2014). For this aim the animals are introduced in treadmills or motor wheels with a gradual increase in training load. This condition may be relevant to improve locomotor performance and minimize potential injures that can occur in this new environment for the rodents. In our study a 100% of rats finished the whole running habituation period. However, the habituation protocol is absent in some studies using forced motor wheel; which maintain a constant intensity and volume from the beginning and throughout all the sessions during their programs (Clement et al., 1993; Ji et al., 2014). The intensity of the exercise developed in these works during the training protocol was between 1.22 m/min and 12 m/min; that is lower in comparison with other forced protocols that includes a progressive increment in the training load, and reaches a maximum speed of 30 m/min (Clement et al., 1993; Leasure and Jones, 2008; Chen et al., 2014; Ji et al., 2014). Some works developed habituation-like protocols, that consist in an increase of training load pattern during the entire training period. Under this conditions the highest speed reached during the training protocol was between 12 m/min and 14 m/min (Sandrow-Feinberg et al., 2009; Caton et al., 2012; Griesbach et al., 2013). However, others works that implement pretraining sessions do not report the training load details of the protocol employed, such us duration, speed or number of sessions; but reported a maximum speed of 21 m/min (O’Dell et al., 2007; Hu et al., 2010; Kennard and Woodruff-Pak, 2012). Finally, only few studies indicate all the detailed features of the habituation protocol developed; reaching the highest speed (30 m/min; Auriat et al., 2006; Shimizu and Yamanouchi, 2011; Wang et al., 2013; Chen et al., 2014). According to Chen et al. (2014), running behavior in motor wheels for rodents is more laborious than the linear motion of the treadmill, in particular in the absence of an adaptive learning stage. Our results demonstrated that habituated rats can sustain a higher forced running speed when compared to the non-habituated group. Interestingly, such locomotor improvement is transient as the forced running speed decreases progressively over time within a period of 30 days.

Several mechanisms could contribute to sustaining a higher running speed following a progressive habituation protocol to the running wheel. It is well known that physical activity produces biochemical changes that improves the muscular erobic metabolism (Terjung and Hood, 1986). While data on how a forced running wheel affects muscular endurance are lacking, evidences from a number of studies using treadmill suggest that a 60 min/day/1 week of running at 25 m/min is needed to induce muscular endurance as determined by changes in cytochrome c, citrate synthase, 3-Ketaocid-CoA transferase (Booth and Holloszy, 1977; Terjung, 1979; Dudley et al., 1982), and myoglobin concentration (Lawrie, 1953; Pattengale and Holloszy, 1967; Terjung and Hood, 1986). In this regard, it is unlikely that 7 days of progressive augmentation in duration and intensity reaching a maximal speed of 9 m/min for 1 h during the last day is sufficient to elicit adaptive changes in muscular endurance. Another contributing factor is stress. Stress hormones are known to increase during forced exercise (Saito and Soya, 2004; Chen et al., 2016), which in turn are increased during acute running according to exercise intensity and duration (Chennaoui et al., 2002; Kawashima et al., 2004; Saito and Soya, 2004; Chen et al., 2016). Acute stress conditions promote fight-fight responses producing elevation of peripheral blood pressure and heart rate; and facilitates energy utilization (Maier et al., 1998; Moraska et al., 2000; Norris and Carr, 2013). However, our habituation protocol reaches a maximal speed of 9 m/min only during the last day for 1 h; and the evidence from a number of studies using treadmill suggest that a running speed of at least 25 m/min (just above the lactate threshold) is required to induce changes in blood lactate, plasma ACTH, plasma glucose and adrenaline (Timofeeva et al., 2003; Saito and Soya, 2004; Soya et al., 2007; Chen et al., 2016). One study showed that activation of CRH neurons in the paraventricular nucleus emerges following 1 h of acute forced running wheel (Yanagita et al., 2007), yet data showing changes in stress hormones are currently lacking.

Based on currently available literature, it is possible that a change in the dopaminergic system may contribute to enhance the forced running-wheel performance observed following the habituation training. It has been shown that pharmacological activation of the dopaminergic system is sufficient to improve motor coordination and its endurance (Tamasy et al., 1981; Freed and Yamamoto, 1985; Boldry et al., 1991; Meeusen and De Meirleir, 1995; Sutoo and Akiyama, 1996; Chen et al., 2016). Future studies are warranted to determine the role of dopamine and related catecholamines in sustaining better locomotor performance in a forced running-wheel system.

Further studies using metabolic chambers or implanted chip systems will be essential to develop the best training condition and standardized exercise training programs in rodents. A rigorous analysis of physiological variations such us VO2max, lactate threshold, heart rate or body composition, a set of data that is currently used in elite sports training in humans, is necessary to adapt the training program to the specific features of each experimental subject (Copp et al., 2009; Zhou et al., 2010).

## Author Contributions

AT and JLF: study conception and design, data collection and analysis, interpretation, drafting and revising the manuscript. RB: data collection and analysis, interpretation, drafting and revising the manuscript. EDC: study conception and design, data collection and analysis, interpretation, and revising the manuscript. NM-D: data collection and analysis, revision of the manuscript. JGP: study conception and design, data collection and analysis, revision of the manuscript. AA: data analysis, interpretation, revision of the manuscript. KYT: study conception and design, data analysis, interpretation, revision of the manuscript. All authors have approved the final manuscript version.

## Funding

Granted by MAPFRE Foundation (2014); and the Spanish Ministry of Science and Technology (MEC) and European Regional Development Fund (FEDER; BFU2014-57516P to JLF).

## Conflict of Interest Statement

The authors declare that the research was conducted in the absence of any commercial or financial relationships that could be construed as a potential conflict of interest.

## References

[B1] Auriat A. M. Grams J. D. Yan R. H. Colbourne F. (2006). Forced exercise does not improve recovery after hemorrhagic stroke in rats. Brain Res. 1109, 183–191. 10.1016/j.brainres.2006.06.035 16854389

[B2] Bentley D. J. Newell J. Bishop D. (2007). Incremental exercise test design and analysis: implications for performance diagnostics in endurance athletes. Sports Med. 37, 575–586. 10.2165/00007256-200737070-00002 17595153

[B4] Boldry R. C. Willins D. L. Wallace L. J. Uretsky N. J. (1991). The role of endogenous dopamine in the hypermotility response to intra-accumbens AMPA. Brain Res. 559, 100–108. 10.1016/0006-8993(91)90292-4 1685936

[B5] Booth F. W. Holloszy J. O. (1977). Cytochrome c turnover in rat skeletal muscles. J. Biol. Chem. 252, 416–419. 188815

[B7] Caton S. J. Bielohuby M. Bai Y. Spangler L. J. Burget L. Pfluger P.. (2012). Low-carbohydrate high-fat diets in combination with daily exercise in rats: effects on body weight regulation, body composition and exercise capacity. Physiol. Behav. 106, 185–192. 10.1016/j.physbeh.2012.02.003 22342194

[B9] Chen C. C. Chang M. W. Chang C. P. Chan S. C. Chang W. Y. Yang C. L.. (2014). A forced running wheel system with a microcontroller that provides high-intensity exercise training in an animal ischemic stroke model. Braz. J. Med. Biol. Res. 47, 858–868. 10.1590/1414-431x20143754 25140816 PMC4181221

[B8] Chen C. Nakagawa S. An Y. Ito K. Kitaichi Y. Kusumi I. (2016). The exercise-glucocorticoid paradox: how exercise is beneficial to cognition, mood, and the brain while increasing glucocorticoid levels. Front. Neuroendocrinol. 44, 83–102. 10.1016/j.yfrne.2016.12.001 27956050

[B10] Chennaoui M. Gomez Merino D. Lesage J. Drogou C. Guezennec C. Y. (2002). Effects of moderate and intensive training on the hypothalamo-pituitary-adrenal axis in rats. Acta Physiol. Scand. 175, 113–121. 10.1046/j.1365-201X.2002.00971.x 12028131

[B11] Clement H. W. Schäfer F. Ruwe C. Gemsa D. Wesemann W. (1993). Stress-induced changes of extracellular 5-hydroxyindoleacetic acid concentrations followed in the nucleus raphe dorsalis and the frontal cortex of the rat. Brain Res. 614, 117–124. 10.1016/0006-8993(93)91024-m 7688645

[B12] Copp S. W. Davis R. T. Poole D. C. Musch T. I. (2009). Reproducibility of endurance capacity and VO2peak in male Sprague-Dawley rats. J. Appl. Physiol. 106, 1072–1078. 10.1152/japplphysiol.91566.2008 19213934

[B13] Cotman C. W. Berchtold N. C. (2002). Exercise: a behavioral intervention to enhance brain health and plasticity. Trends Neurosci. 25, 295–301. 10.1016/s0166-2236(02)02143-4 12086747

[B14] Creer D. J. Romberg C. Saksida L. M. van Praag H. Bussey T. J. (2010). Running enhances spatial pattern separation in mice. Proc. Natl. Acad. Sci. U S A 107, 2367–2372. 10.1073/pnas.0911725107 20133882 PMC2836679

[B15] Dick F. W. (2007). Sports Training Principles. An Introduction to Sports Science. 6th Edn. New York, NY: Bloomsbury Sport.

[B17] Dishman R. K. Berthoud H. R. Booth F. W. Cotman C. W. Edgerton V. R. Fleshner M. R.. (2006). Neurobiology of exercise. Obesity 14, 345–356. 10.1038/oby.2006.46 16648603

[B16] Dishman R. Heath G. Lee I. M. (2013). Physical Activity Epidemiology. Champaign, IL: Human Kinetics.

[B18] Dudley G. A. Abraham W. M. Terjung R. L. (1982). Influence of exercise intensity and duration on biochemical adaptations in skeletal muscle. J. Appl. Physiol. Respir. Environ. Exerc. Physiol. 53, 844–850. 6295989 10.1152/jappl.1982.53.4.844

[B19] Edgerton V. R. Tillakaratne N. J. Bigbee A. J. de Leon R. D. Roy R. R. (2004). Plasticity of the spinal neural circuitry after injury. Annu. Rev. Neurosci. 27, 145–167. 10.1146/annurev.neuro.27.070203.144308 15217329

[B20] Freed C. R. Yamamoto B. K. (1985). Regional brain dopamine metabolism: a marker for the speed, direction, and posture of moving animals. Science 229, 62–65. 10.1126/science.4012312 4012312

[B21] Griesbach G. S. Tio D. L. Nair S. Hovda D. A. (2013). Temperature and heart rate responses to exercise following mild traumatic brain injury. J. Neurotrauma 30, 281–291. 10.1089/neu.2012.2616 23009619 PMC3579384

[B22] Hillman C. H. Erickson K. I. Kramer A. F. (2008). Be smart, exercise your heart: exercise effects on brain and cognition. Nat. Rev. Neurosci. 9, 58–65. 10.1038/nrn2298 18094706

[B23] Hu X. Zheng H. Yan T. Pan S. Fang J. Jiang R.. (2010). Physical exercise induces expression of CD31 and facilitates neural function recovery in rats with focal cerebral infarction. Neurol. Res. 32, 397–402. 10.1179/016164110X12670144526309 20483007

[B24] Jasperse J. L. Laughlin M. H. (1999). Vasomotor responses of soleus feed arteries from sedentary and exercise-trained rats. J. Appl. Physiol. 86, 441–449. 9931174 10.1152/jappl.1999.86.2.441

[B25] Ji J. F. Ji S. J. Sun R. Li K. Zhang Y. Zhang L. Y.. (2014). Forced running exercise attenuates hippocampal neurogenesis impairment and the neurocognitive deficits induced by whole-brain irradiation via the BDNF-mediated pathway. Biochem. Biophys. Res. Commun. 443, 646–651. 10.1016/j.bbrc.2013.12.031 24333433

[B26] Kawashima H. Saito T. Yoshizato H. Fujikawa T. Sato Y. McEwen B. S.. (2004). Endurance treadmill training in rats alters CRH activity in the hypothalamic paraventricular nucleus at rest and during acute running according to its period. Life Sci. 76, 763–774. 10.1016/j.lfs.2004.09.014 15581908

[B27] Kennard J. A. Woodruff-Pak D. S. (2012). A comparison of low-and high-impact forced exercise: effects of training paradigm on learning and memory. Physiol. Behav. 106, 423–427. 10.1016/j.physbeh.2012.02.023 22402029 PMC3349001

[B28] Kobilo T. Liu Q.-R. Gandhi K. Mughal M. Shaham Y. van Praag H. (2011). Running is the neurogenic and neurotrophic stimulus in environmental enrichment. Learn. Mem. 18, 605–609. 10.1101/lm.2283011 21878528 PMC3166785

[B29] Koch L. G. Britton S. L. (2001). Artificial selection for intrinsic aerobic endurance running capacity in rats. Physiol. Genomics 5, 45–52. 11161005 10.1152/physiolgenomics.2001.5.1.45

[B30] Kregel K. C. Allen D. L. Booth F. W. Fleshner M. R. Henriksen E. J. Musch T. I. (2006). Resource Book for the Design of Animal Exercise Protocols. Bethesda, MD: American Physiological Society.

[B31] Lawrie R. A. (1953). Effect of enforced exercise on myoglobin concentration in muscle. Nature 171, 1069–1070. 10.1038/1711069a0 13063531

[B33] Leasure J. L. Jones M. (2008). Forced and voluntary exercise differentially affect brain and behavior. Neuroscience 156, 456–465. 10.1016/j.neuroscience.2008.07.041 18721864

[B34] Maier S. F. Fleshner M. Watkins L. R. (1998). “Neural, endocrine, and immune mechanisms of stress-induced immunomodulation,” in New Frontiers in Stress Research: Modulation of Brain Function, eds Levy A. Grauer E. Ben-Nathan D. de Kloet Chur E. R. (Switzerland: Harwood Academic), 117–126.

[B35] Marlatt M. W. Potter M. C. Lucassen P. J. van Praag H. (2012). Running throughout middle-age improves memory function, hippocampal neurogenesis and BDNF levels in female C57BL/6J mice. Dev. Neurobiol. 72, 943–952. 10.1002/dneu.22009 22252978 PMC3485396

[B36] Meeusen R. De Meirleir K. (1995). Exercise and brain neurotransmission. Sports Med. 20, 160–188. 10.2165/00007256-199520030-00004 8571000

[B37] Mora-Rodriguez R. Ortega J. F. Guio de Prada V. Fernández-Elías V. E. Hamouti N. Morales-Palomo F.. (2016). Effects of simultaneous or sequential weight loss diet and aerobic interval training on metabolic syndrome. Int. J. Sports Med. 37, 274–281. 10.1055/s-0035-1564259 26667921

[B38] Moraska A. Deak T. Spencer R. L. Roth D. Fleshner M. (2000). Treadmill running produces both positive and negative physiological adaptations in Sprague-Dawley rats. Am. J. Physiol. Regul. Integr. Comp. Physiol. 279, R1321–R1329. 11004000 10.1152/ajpregu.2000.279.4.R1321

[B39] Narath E. Skalicky M. Viidik A. (2001). Voluntary and forced exercise influence the survival and body composition of ageing male rats differently. Exp. Gerontol. 36, 1699–1711. 10.1016/s0531-5565(01)00145-0 11672990

[B40] Norris D. O. Carr J. A. (2013). Vertebrate Endocrinology. Waltham, MA: Academic Press.

[B41] O’Dell S. J. Gross N. B. Fricks A. N. Casiano B. D. Nguyen T. B. Marshall J. F. (2007). Running wheel exercise enhances recovery from nigrostriatal dopamine injury without inducing neuroprotection. Neuroscience 144, 1141–1151. 10.1016/j.neuroscience.2006.10.042 17157992

[B42] Pattengale P. K. Holloszy J. O. (1967). Augmentation of skeletal muscle myoglobin by a program of treadmill running. Am. J. Physiol. 213, 783–785. 6036801 10.1152/ajplegacy.1967.213.3.783

[B43] Saito T. Soya H. (2004). Delineation of responsive AVP-containing neurons to running stress in the hypothalamus. Am. J. Physiol. Regul. Integr. Comp. Physiol. 286, R484–R490. 10.1152/ajpregu.00453.2003 14630623

[B44] Sandrow-Feinberg H. R. Izzi J. Shumsky J. S. Zhukareva V. Houle J. D. (2009). Forced exercise as a rehabilitation strategy after unilateral cervical spinal cord contusion injury. J. Neurotrauma 26, 721–731. 10.1089/neu.2008.0750 19489718 PMC2848827

[B46] Shimizu H. Yamanouchi K. (2011). Acceleration of irregular estrous cycle in forced running by midbrain raphe lesions in female rats. Neurosci. Lett. 495, 192–195. 10.1016/j.neulet.2011.03.063 21457760

[B47] Smith A. D. Zigmond M. J. (2003). Can the brain be protected through exercise? Lessons from an animal model of parkinsonism. Exp. Neurol. 184, 31–39. 10.1016/j.expneurol.2003.08.017 14637076

[B48] Soya H. Mukai A. Deocaris C. C. Ohiwa N. Chang H. Nishijima T.. (2007). Threshold-like pattern of neuronal activation in the hypothalamus during treadmill running: establishment of a minimum running stress (MRS) rat model. Neurosci. Res. 58, 341–348. 10.1016/j.neures.2007.04.004 17524508

[B49] Stummer W. Weber K. Tranmer B. Baethmann A. Kempski O. (1994). Reduced mortality and brain damage after locomotor activity in gerbil forebrain ischemia. Stroke 25, 1862–1869. 10.1161/01.STR.25.9.1862 8073470

[B50] Sutoo D. E. Akiyama K. (1996). The mechanism by which exercise modifies brain function. Physiol. Behav. 60, 177–181. 10.1016/0031-9384(96)00011-x 8804660

[B51] Tamasy V. Koranyi L. Phelps C. P. (1981). The role of dopaminergic and serotonergic mechanisms in the development of swimming ability of young rats. Dev. Neurosci. 4, 389–400. 10.1159/000112778 7327101

[B52] Tchernichovski O. Benjamini Y. (1998). The dynamics of long term exploration in the rat: part II. An analytical model of the kinematic structure of rat exploratory behavior. Biol. Cybern. 78, 433–440. 10.1007/s004220050447 9711817

[B53] Terjung R. L. (1979). The turnover of cytochrome c in different skeletal-muscle fibre types of the rat. Biochem. J. 178, 569–574. 10.1042/bj1780569 222256 PMC1186555

[B54] Terjung R. L. Hood D. A. (1986). Biochemical adaptations in skeletal muscle induced by exercise training. ACS Symp. Ser. Am. Chem. Soc. 294, 8–26. 10.1021/bk-1986-0294.ch002

[B55] Timofeeva E. Huang Q. Richard D. (2003). Effects of treadmill running on brain activation and the corticotropin-releasing hormone system. Neuroendocrinologyy 77, 388–405. 10.1159/000071311 12845225

[B56] van Praag H. (2009). Exercise and the brain: something to chew on. Trends Neurosci. 32, 283–290. 10.1016/j.tins.2008.12.007 19349082 PMC2680508

[B57] van Praag H. Shubert T. Zhao C. Gage F. H. (2005). Exercise enhances learning and hippocampal neurogenesis in aged mice. J. Neurosci. 25, 8680–8685. 10.1523/JNEUROSCI.1731-05.2005 16177036 PMC1360197

[B58] Vivar C. Potter M. C. van Praag H. (2012). “All about running: synaptic plasticity, growth factors and adult hippocampal neurogenesis,” in Neurogenesis and Neural Plasticity, eds Belzung C. Wigmore P. (Berlin, Heidelberg: Springer), 189–210. 10.1007/7854_2012_220 PMC4565722 22847651

[B59] Wang Z. Myers K. G. Guo Y. Ocampo M. A. Pang R. D. Jakowec M. W.. (2013). Functional reorganization of motor and limbic circuits after exercise training in a rat model of bilateral parkinsonism. PLoS One 8:e80058. 10.1371/journal.pone.0080058 24278239 PMC3836982

[B60] World Health Organization (2010). Global Recommendations on Physical Activity for Health. Switzerland: World Health Organization. 26180873

[B61] Yanagita S. Amemiya S. Suzuki S. Kita I. (2007). Effects of spontaneous and forced running on activation of hypothalamic corticotropin-releasing hormone neurons in rats. Life Sci. 80, 356–363. 10.1016/j.lfs.2006.09.027 17067638

[B62] Zhou Y. Yuan Y. Gao J. Yang L. Zhang F. Zhu G.. (2010). An implanted closed-loop chip system for heart rate control: system design and effects in conscious rats. J. Biomed. Res. 24, 107–114. 10.1016/S1674-8301(10)60018-8 23554620 PMC3596544

